# Genome-wide analysis of UDP-glycosyltransferases family and identification of UGT genes involved in drought stress of *Platycodon grandiflorus*

**DOI:** 10.3389/fpls.2024.1363251

**Published:** 2024-04-29

**Authors:** Bowen Chen, Xinrui Wang, Hanwen Yu, Nan Dong, Jing Li, Xiangwei Chang, Jutao Wang, Chao Jiang, Juan Liu, Xiulian Chi, Liangping Zha, Shuangying Gui

**Affiliations:** ^1^ College of Pharmacy, Anhui University of Chinese Medicine, Hefei, China; ^2^ Center for Xin’an Medicine and Modernization of Traditional Chinese Medicine of IHM, Anhui University of Chinese Medicine, Hefei, China; ^3^ State Key Laboratory of Dao-Di Herbs, National Resource Center for Chinese Materia Medica, China Academy of Chinese Medical Sciences, Beijing, China; ^4^ Chinese Academy of Medical Sciences Research Unit (No. 2019RU057), National Resource Center for Chinese Materia Medica, China Academy of Chinese Medical Sciences, Beijing, China; ^5^ Institute of Conservation and Development of Traditional Chinese Medicine Resources, Anhui Academy of Chinese Medicine, Hefei, China; ^6^ Anhui Province Key Laboratory of Research and Development of Chinese Medicine, Hefei, China; ^7^ Institute of Pharmaceutics, Anhui Academy of Chinese Medicine, Hefei, China; ^8^ Anhui Province Key Laboratory of Pharmaceutical Technology and Application Anhui University of Chinese Medicine, Hefei, China; ^9^ MOE-Anhui Joint Collaborative Innovation Center for Quality Improvement of Anhui Genuine Chinese Medicinal Materials, Hefei, China

**Keywords:** *Platycodon grandiflorus*, UDP-glycosyltransferase, genome-wide identification, expression analysis, drought stress

## Abstract

**Introduction:**

The uridine diphosphate (UDP)-glycosyltransferase (UGT) family is the largest glycosyltransferase family, which is involved in the biosynthesis of natural plant products and response to abiotic stress. UGT has been studied in many medicinal plants, but there are few reports on Platycodon grandiflorus. This study is devoted to genome-wide analysis of UGT family and identification of UGT genes involved in drought stress of Platycodon grandiflorus (PgUGTs).

**Methods:**

The genome data of Platycodon grandiflorus was used for genome-wide identification of PgUGTs, online website and bioinformatics analysis software was used to conduct bioinformatics analysis of PgUGT genes and the genes highly responsive to drought stress were screened out by qRT-PCR, these genes were cloned and conducted bioinformatics analysis.

**Results:**

A total of 75 PgUGT genes were identified in P.grandiflorus genome and clustered into 14 subgroups. The PgUGTs were distributed on nine chromosomes, containing multiple cis-acting elements and 22 pairs of duplicate genes were identified. Protein-protein interaction analysis was performed to predict the interaction between PgUGT proteins. Additionally, six genes were upregulated after 3d under drought stress and three genes (PGrchr09G0563, PGrchr06G0523, PGrchr06G1266) responded significantly to drought stress, as confirmed by qRT-PCR. This was especially true for PGrchr06G1266, the expression of which increased 16.21-fold after 3d of treatment. We cloned and conducted bioinformatics analysis of three candidate genes, both of which contained conserved motifs and several cis-acting elements related to stress response, PGrchr06G1266 contained the most elements.

**Discussion:**

PgGT1 was confirmed to catalyze the C-3 position of platycodin D and only eight amino acids showed differences between gene PGr008G1527 and PgGT1, which means PGr008G1527 may be able to catalyze the C-3 position of platycodin D in the same manner as PgGT1. Seven genes were highly expressed in the roots, stems, and leaves, these genes may play important roles in the development of the roots, stems, and leaves of P. grandiflorus. Three genes were highly responsive to drought stress, among which the expression of PGrchr06G1266 was increased 16.21-fold after 3d of drought stress treatment, indicating that PGrchr06G1266 plays an important role in drought stress tolerance. To summarize, this study laied the foundation to better understand the molecular bases of responses to drought stress and the biosynthesis of platycodin.

## Introduction

Glycosylation is a universal modification in plants. It is important for secondary metabolite production, defense, and abiotic stress resistance (Khorolragchaa et al., 2014; Lamichhaney et al., 2017). Uridine diphosphate glycosyltransferase (UGT), with a conserved PSPG-box (Vogt and Jones, 2000), is the largest glycosyltransferase family in plant species (Yang et al., 2020). It promotes glycosylation by catalyzing the transfer of sugar molecules from activated donor molecules to specific receptors (Wu et al., 2017). UGTs are involved in the biosynthesis of natural plant products such as flavonoids (Dai et al., 2017; Yin et al., 2017), phenylpropanoids (Lin et al., 2016), and terpenoids (Zheng et al., 2019; Zhao et al., 2020), and also introduce sugar groups at specific positions on different substrates to produce saponins (Brazier-Hicks et al., 2018).

To date, UGTs have been identified and extensively studied across many taxa, including bacteria, fungi, animals, plants, and viruses (Campbell et al., 1997). UGTs were first found in maize (Dooner and Nelson, 1977), after which they were identified in species ranging from lower to higher plants, including 182 examples in soybean (Mamoon Rehman et al., 2016), 241 in apple (Velasco et al., 2010), and 145 in pomelo (Wu et al., 2020). Additionally, previous studies have shown that several UGT genes play notable roles in stress resistance. MdUGT83L3 in *Malus domestica* modulates flavonoid metabolism to adapt to salt and cold stress (Li et al., 2022c). Several NtUGT genes were identified to play distinct roles in cold and drought resistance (Yang et al., 2023). MsUGT003 and MsUGT024 responded significantly to drought stress and ABA treatments (Ao et al., 2022). Overexpression of PhUGT51 significantly increased the salt tolerance of *Petunia hybrida* (Dong et al., 2022) and AtUGT85A5 also responded to salt stress (Sun et al., 2013). Therefore, these genes may help the plants to adapt to changing environments.

The dried root of *Platycodon grandiflorus* (Jacq.) A.DC are commonly used in traditional Chinese medicine and it has a wide range of pharmacological effects, including anti-inflammatory (Qu et al., 2016; Li et al., 2022a), antioxidant (Wang et al., 2019a; Ma et al., 2021), antitumor (Zeng et al., 2016; Li et al., 2019), immunoregulatory (Zhao et al., 2017; Wang et al., 2019b), hypotensive (Lin et al., 2017), hypolipidemic (Hwang et al., 2013; Kim et al., 2019), hypoglycemic (Kim et al., 2000), and hepatoprotective (Leng et al., 2018; Liu et al., 2020) effects. Platycodin, as the main active compounds of *P. grandiflorus*, possesses potent pharmacological effects, so it is necessary to reveal the genes involved in the biosynthesis of platycodin. Drought stress greatly impacts the morphological, physiological, and molecular characteristics of plants (Abreha et al., 2021) as well as the accumulation of metabolites. Notably, researchers have confirmed that the platycodin content in *P. grandiflorus* roots increases under short-term drought stress (Li et al., 2022b). Our research group detected the platycodin content in *P. grandiflorus* under drought stress, finding that it promotes the accumulation of platycodin content to a certain extent (Yu et al., 2024). UGT gene is the key downstream gene in the biosynthesis of platycodin. It can be processed through oxidation and glycosylation to produce different types of platycodin, playing an important role in the biosynthesis of platycodin. Hence, we chose drought stressed *P. grandiflorus* as the material to observe which UGT genes were significantly upregulated under drought stress. We speculate that these upregulated UGT genes are likely to be involved in the biosynthesis of platycodin and are the key genes that lead to increased platycodin content.

In the present study, we identified 75 PgUGTs clustered into 14 subgroups. The chromosome locations, structural analysis, *cis*-acting elements, intraspecific collinearity analysis and protein-protein interaction analysis of the PgUGTs were conducted with using web tools. The expression patterns of PgUGTs in roots, stems, and leaves and responses to drought stress were investigated, with PgUGTs showing tissue specificity and involvement in the drought stress response. We identified three UGT genes that respond highly to drought stress and speculated that these may be the key genes in platycodin biosynthesis. This study is expected to provide better understanding of the mechanisms of PgUGTs in the drought stress resistance process and provide reference for further research on the biosynthesis of platycodin.

## Materials and methods

### Identification of PgUGTs

The sequences identified as UGT genes with a PSPG-box were downloaded from NCBI and the BLAST tool in the BioEdit software was used to build a database of the *P. grandiflorus* genome; the downloaded UGT sequences were used as a probe to search. In the local BLAST process, the E value was set to 1.0E^-50^, max number of hits to report was set to 500, max number of alignments to show was set to 250, threshold for extending hit was set to 0, and the matrix was set BLOSUM62. Additionally, based on the KEGG annotation results of genomic data, we retained the genes annotated as UDP-glycosyltransferase. The Pfam (http://pfam.sanger.ac.uk/search) and NCBI Conserved Domain Search (https://www.ncbi.nlm.nih.gov/Structure/cdd/wrpsb.cgi) were used to verify the structural domains of each candidate gene, and retained genes with the correct domain “Glycosyltransferase_GTB” type superfamily. The online software ExPASy (https://www.expasy.org/) was used to analyze the number of amino acids, molecular weights, and isoelectric points of the screened UGT. The online software Cell-PLoc (http://www.csbio.sjtu.edu.cn) was used to predict subcellular localization.

### Phylogenetic analysis of PgUGTs

MEGA software was used to build the tree using the neighbor-joining method (Bootstrap value was set to 1000). The online software iTOL (https://itol.embl.de/index.shtml) was used to modify the tree.

### Chromosomal location of PgUGTs

The corresponding chromosome of each UGT was determined based on genomic GFF data of *P. grandiflorus*. “Gene Location Visualize” from the GTF/GFF function in TBtools software was used to complete the visual drawing.

### Structural analysis of PgUGTs

Based on the GFF file and amino acid sequence of the PgUGTs genome, the gene structure and intron distribution of PgUGTs were visualized using the Gene Structure View function in TBtools (Chen et al., 2020). MEME software was used to analyze its conserved motif, the predictive value was set to 10, and TBtools was used for visual analysis.

### Analysis of cis-acting elements in PgUGTs promoter regions

The upstream 3000 bp promoter sequence of PgUGTs were input into PlantCARE (https://bioinformatics.psb.ugent.be/webtools/plantcare/html), and the *cis*-acting element of the promoter was analyzed through this software. The “Basic Biosequence View” function in TBtools was used to complete the visual drawing.

### Intraspecific collinearity analysis of PgUGTs

A whole-genome collinearity analysis of PgUGTs was performed based on the genomic data of *P. grandiflorus*. We first align the protein sequences within the species to generate a Tab file, then we processed the genome GFF file and imported both files into “Quick MCScanX Wrapper” to generate tandem and collinearity file, tandem file was used to visualize tandemly duplicated genes and collinearity file was used to visualize segmentally duplicated genes.

### Prediction and analysis of PgUGTs member protein interaction

The STRING database (https://cn.string-db.org/) was used to predict AtUGT protein-protein interaction networks. Medium confidence was set 0.400. AtUGT proteins with interactions were mapped to PgUGT proteins by homology.

### Analysis of PgUGTs expression patterns in roots, stems, and leaves

The expression of UGTs in the roots, stems, and leaves of *P. grandiflorus* was analyzed using transcriptome-linked genome data. The “Heatmap” function was used to complete the visualized heat map. To verify the expression pattern of the selected 12 genes in roots, stems, and leaves, total RNA was extracted using the RNA TRIzol kit (Huayueyang Biochemical Technology, Beijing, China), while reverse transcription was performed using the PrimeScript II 1st Strand cDNA Synthesis Kit (Lablead Company, Beijing, China) for cDNA synthesis. A Green qPCR Premix fluorescent quantitative kit (Lablead Company, Beijing, China) was used to conduct real-time, fluorescence-based quantitative PCR.

### Expression analysis under drought stress of PgUGTs

A 15% PEG 6000 solution was used to simulate a dry environment. The material used in this study was fresh biennial *P. grandiflorus* root from Tongcheng, Anhui Province. This experiment added 15% PEG 6000 to liquid culture medium, and drought stress was simulated for 2 and 3d using tissue cultured seedlings in the culture medium. The “Heatmap” function was used to complete the visualized heat map and six genes were selected for quantitative PCR.

### Cloning and bioinformatics analysis of PGrchr06G0523, PGrchr06G1266, and PGrchr09G0563

The pEASY-Blunt Zero Cloning Kit (Transgenbiotech Company, Beijing, China) was used to clone PGrchr06G0523 and PGrchr06G1266, PGrchr09G0563 was sent to total gene synthesis (Jinweizhi Company, Jiangsu, China). The PCR conditions were as follows: 95°C for 2 min; 95°C for 20 s, 56°C for 20 s, and 72°C for 1 min for 40 cycles; the final extension was at 72°C for 5 min. Cut and recycle PCR products and convert it into E. coli Trans-T1 competent cells and then perform bacterial liquid PCR. The bacterial liquid PCR reaction system is 94°C for 5 min; 94°C for 30 s, 55°C for 30 s, 72°C for 1 min for 35 cycles; the final extension was at 72°C for 10 min. Bacterial liquids with purposeful bands were sent for sequencing (Tongyongbiotech Company, Anhui, China). The three-dimensional structure of candidate UGTs were built on SWISS-MODEL (https://swissmodel.expasy.org/) and the Pymol system was used to modify them. The upstream 1500 bp promoter sequence of the three genes were input into PlantCARE and the Basic Biosequence View function in TBtools was used to complete the visual drawing.

## Results

### Identification of PgUGTs

A total of 75 PgUGTs were identified, and their physical and chemical properties were predicted. These UGT genes encoded predicted proteins ranging from 222–1000 amino acids (average 474.14 amino acids). The molecular weight ranged from 24.5–112.5 kDa (average Mw = 53.08 kDa) and the isoelectric point ranged from 4.79–7.69 (average pI = 5.70). The subcellular localization of these genes indicated that most members were probably in the cell membrane and chloroplasts, while only a few members were in the cytoplasm, extracellular area, mitochondria, nucleus, vacuole, and peroxisomes (Physical and chemical properties of PgUGTs are listed in **Supplementary Table S1**).

### Phylogenetic analysis of PgUGTs

**Figure 1:**
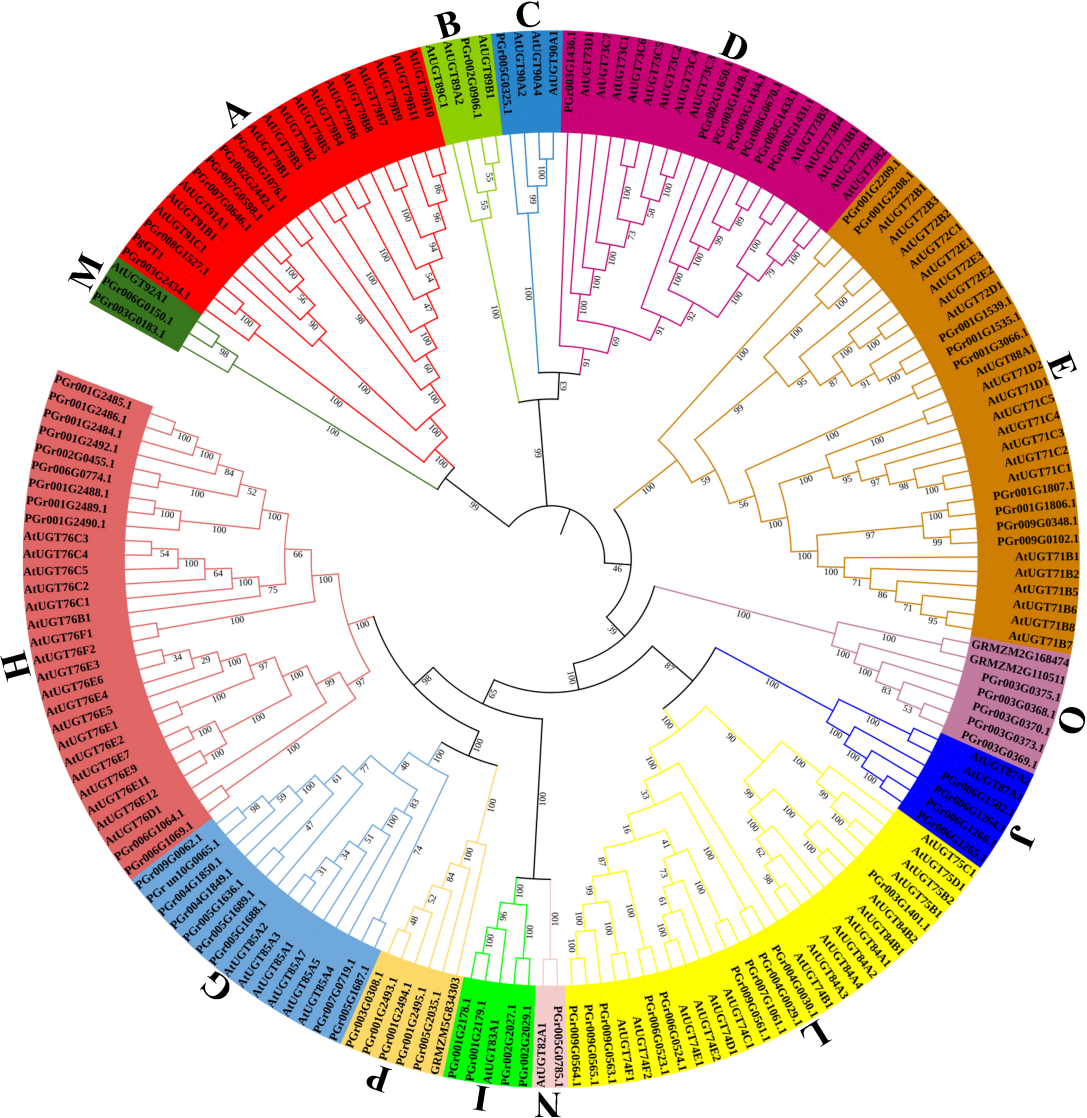
Phylogenetic analysis of PgUGTs and AtUGTs. 75 PgUGTs, 102 AtUGTs, 3 UGT genes in *Zea mays* and PgGT1 in *P. grandiflorus* were selected to construct the tree, and 75 PgUGTs were divided into 14 groups. PgGT1 was marked with a green circle shape, PGr003G2434 and PGr008G1527 were marked with blue star shape.

The phylogenetic tree was constructed using the 75 PgUGTs, 102 AtUGTs, 3 UGT genes in *Zea mays* (Li et al., 2014) (GRMZM5G834303 in group P, GRMZM2G110511 and GRMZM2G168474 in group O) and PgGT1 in *P. grandiflorus* (Tang et al., 2023) (**Figure 1**). Based on the 14 conserved groups (A–N) found in *Arabidopsis thaliana* (Li et al., 2001) and 3 UGT genes in *Zea mays*, the 75 PgUGTs were divided into 14 groups: none were placed in the F and K Groups, while 5 members were in Groups P and O, respectively. Group H was the largest with 11 members, while 10 members were in Group L, 9 members in Groups E and G, 7 members in group D, 6 members in Group A, 4 members in Groups I and J, 2 members in Group M, only one member each in Groups B, C, and N. Members of Groups H, L, E, and G accounted for 52% of all the members, indicating that these four groups were the main components of the PgUGT family. PgGT1 (Tang et al., 2023) was confirmed to have strong functions in *P. grandiflorus*. It was also found to be in Group A and was clustered with PGr008G1527 and PGr003G2434. We compared the amino acids of PgGT1 (GenBank No. OP626755) and PGr008G1527 and found that only eight amino acids showed differences (**Supplementary Figure S1**).

### Chromosomal location of PgUGTs

**Figure 2:**
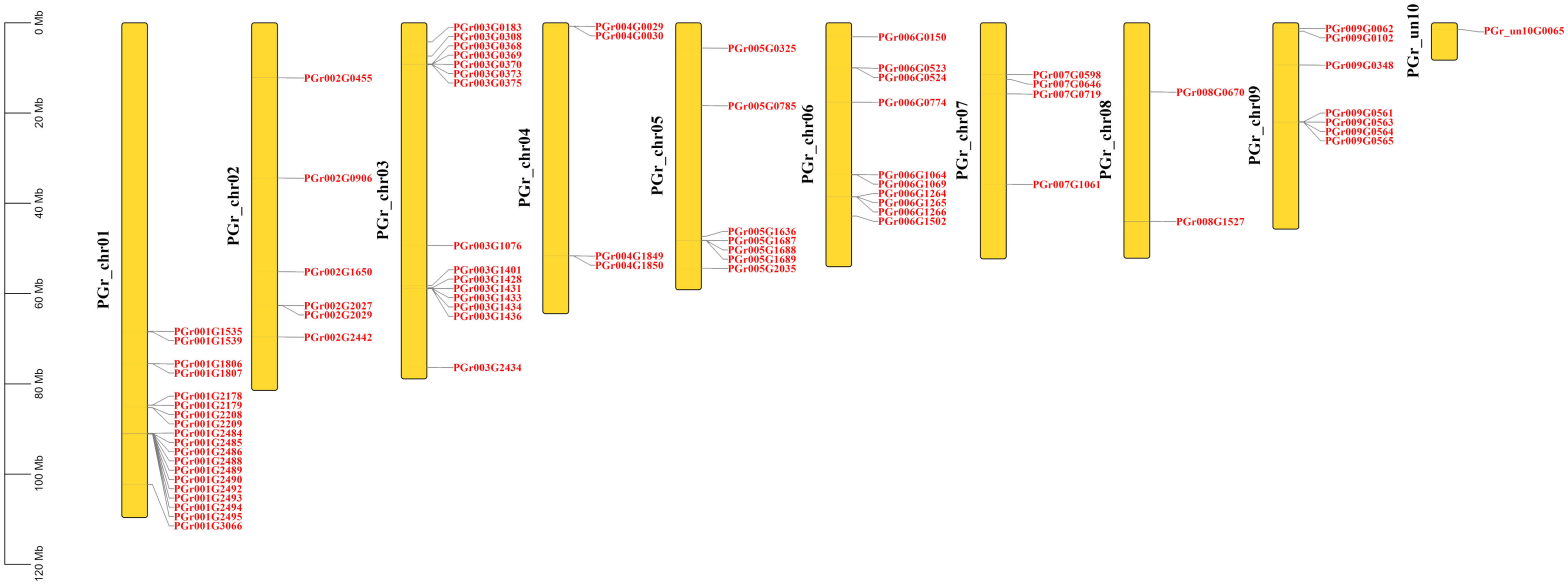
Chromosome distribution of PgUGTs.

A total of 75 PgUGTs were distributed on 9 chromosomes (**Figure 2**). Although chrun10 was not attached to a chromosome, we found one gene on chrun10. Here, we also conducted chromosomal location and other bioinformatic analyses on this gene. Chr01, chr03, and chr06 possessed the highest numbers of UGTs (19, 15, and 10, respectively), whereas chr04, chr07 had four UGTs, only two numbers on chr08.

### Structural analysis of PgUGTs

**Figure 3:**
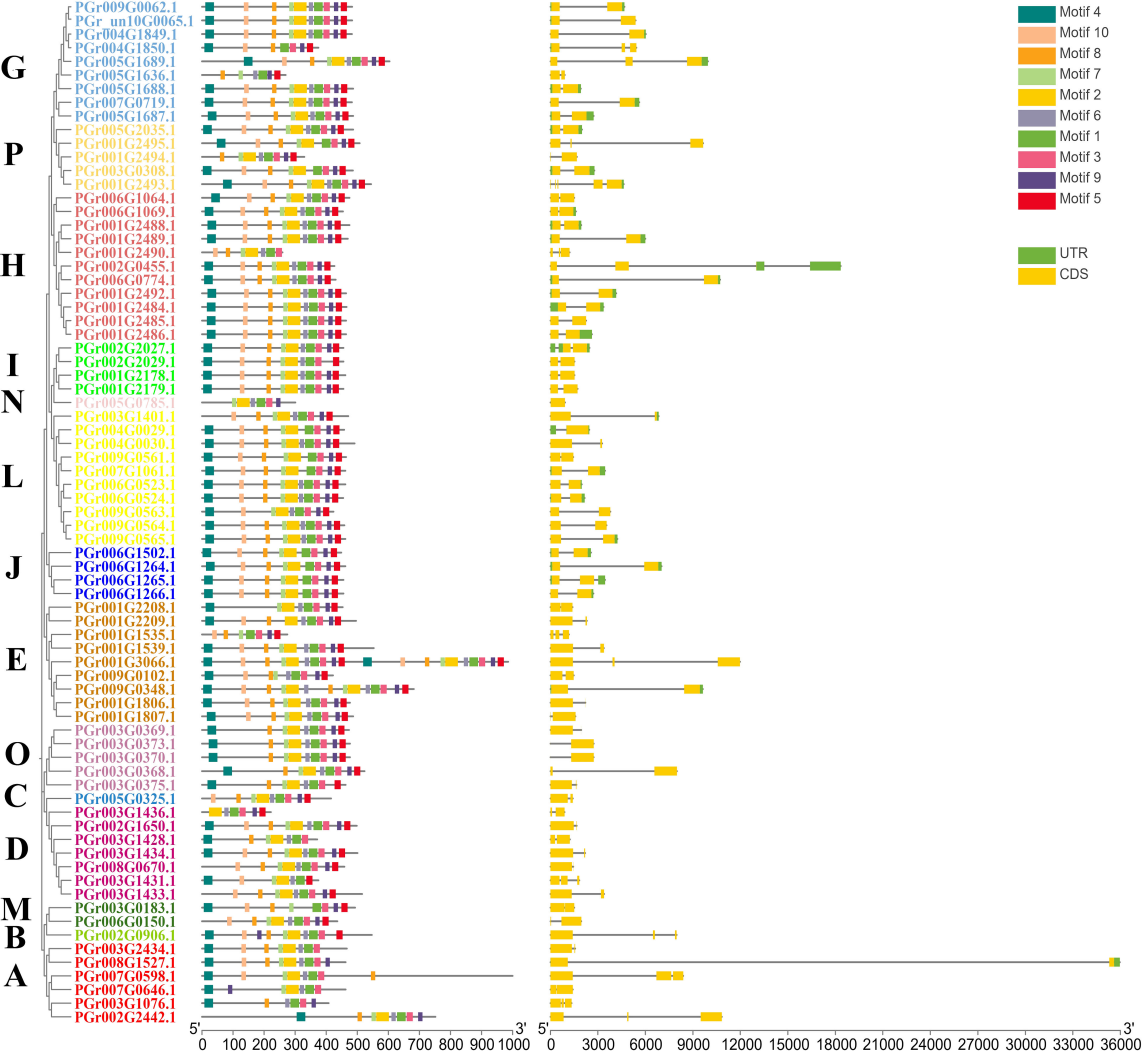
The gene structure of PgUGTs.

Of the 75 UGTs, 74 genes contained introns (**Figure 3**). Introns were divided into three types based on their phase: phase 0, phase 1, and phase 2 (Barvkar et al., 2012). 73, 17, and 42 members contained phases 0, 1, and 2, respectively. Differences in intron locations could provide a basis for further investigation of the biological functions of each member. The PgUGTs contained a conserved motif named PSPG-box (**Supplementary Figure S2**) and members of the same group showed similarities in the distribution of their conserved motifs. For example, among the nine members of Group G, seven members contained identical motifs. Among the 11 members of Group H, 10 contained identical motifs, indicating that the distribution of motifs in the members of the same group was similar to some extent, which may also lead to similar functions.

### Analysis of cis-acting elements in PgUGTs promoter regions

**Figure 4:**
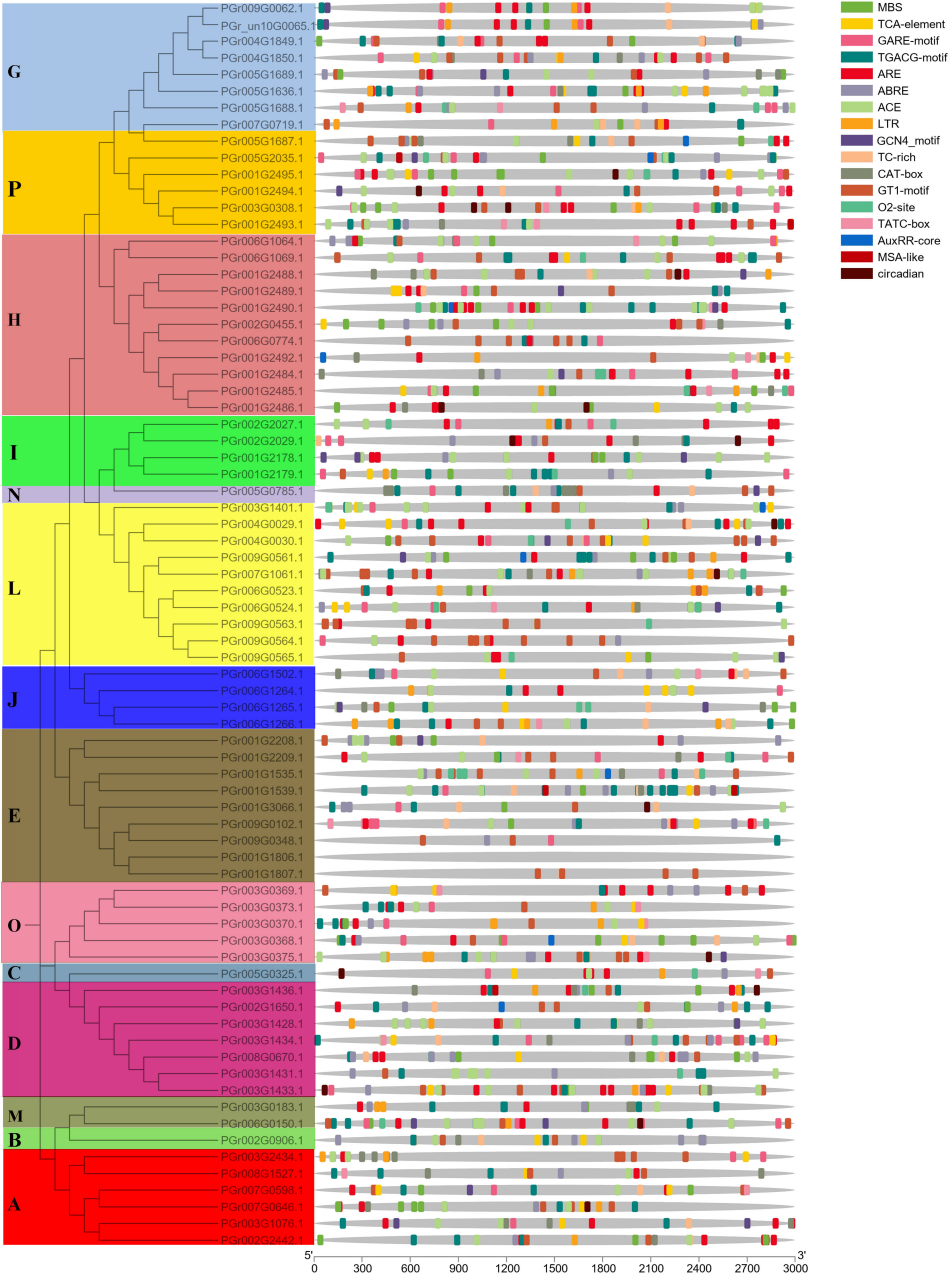
*Cis*-element analysis showed the upstream 3,000 bp promoter sequence of PgUGTs.

The PgUGT promoter region contained many response elements, including defense and stress responses (TC-rich repeats), gibberellin response (TATC-box), light response (ACE), low-temperature response (LTR), salicylic acid response (TCA-element), abscisic acid response (ABRE), auxin response (TGA-element), MeJA-response (CGTCA-motif), drought-inducibility (MBS), and other responses (**Figure 4**). Most genes contained light response and hormone response elements: 35 genes contained low-temperature response elements, 45 genes contained drought-inducibility elements, 31 genes contained defense and stress response elements, indicating that they may play important roles in regulating plant response to stress.

### Intraspecific collinearity analysis of PgUGTs

**Figure 5:**
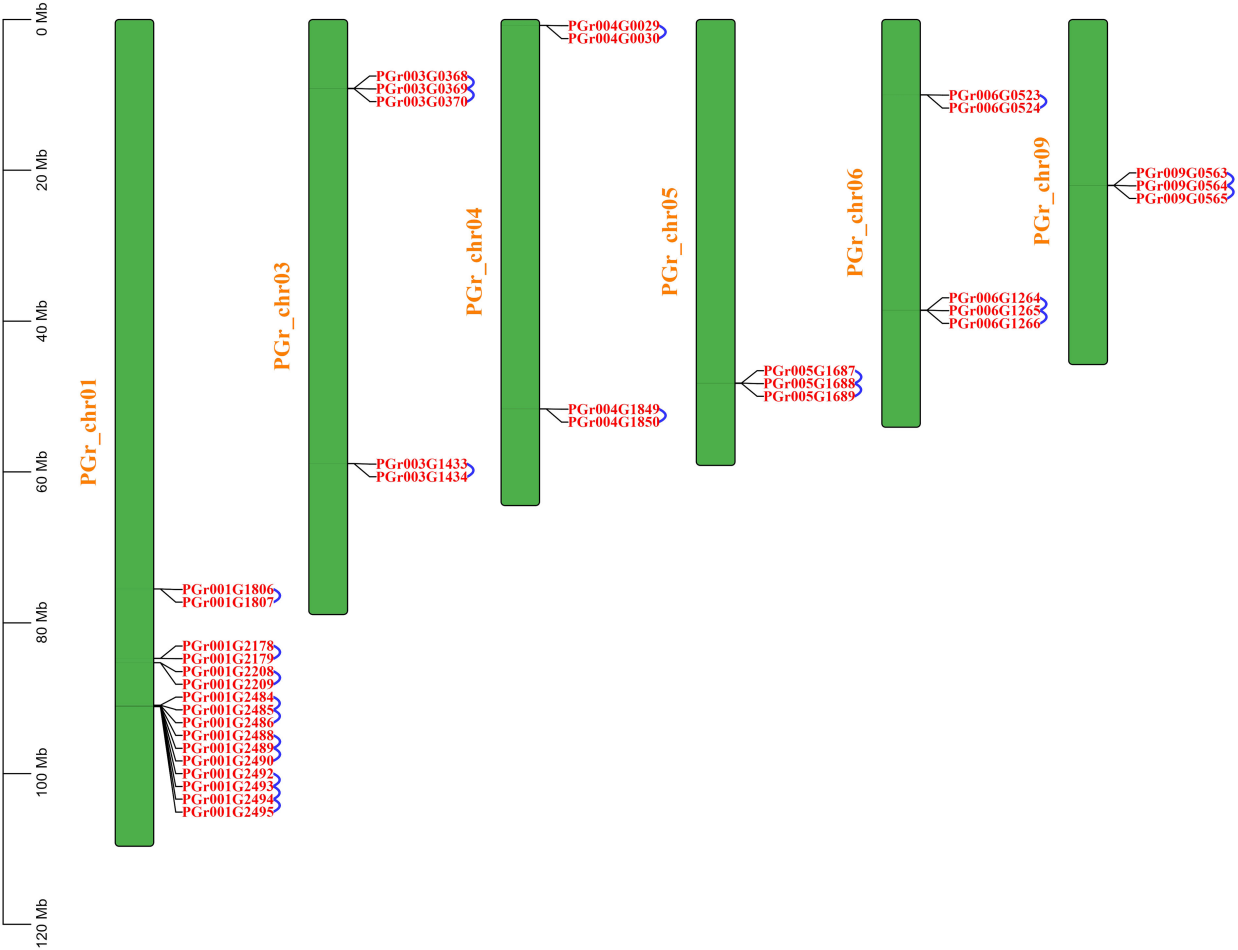
Intraspecific collinearity analysis of PgUGTs. The blue curve represents a pair of tandemly duplicated genes.

22 pairs of tandemly duplicated genes were identified (**Figure 5**), there were no segmentally duplicated genes. 10 pairs of tandemly duplicated genes on Chr01, 3 pairs on Chr03 and Chr06, 2 pairs on Chr04, Chr05 and Chr09, respectively. We conducted amino acid comparisons on the 22 pairs of tandemly duplicated genes (The homology of 22 pairs of tandemly duplicated genes in **Supplementary Table S2**), the sequence consistency of 9 pairs of genes were higher than 70%, among which the sequence consistency of PGr003G0369 and PGr003G0370, PGr006G1265 and PGr006G1266, PGr009G0564 and PGr009G0565 were higher than 90%, these three pairs of genes had a high sequence similarity, we assumed that their functions may be analogous to some extent.

### Prediction and analysis of PgUGT member protein interaction

**Figure 6:**
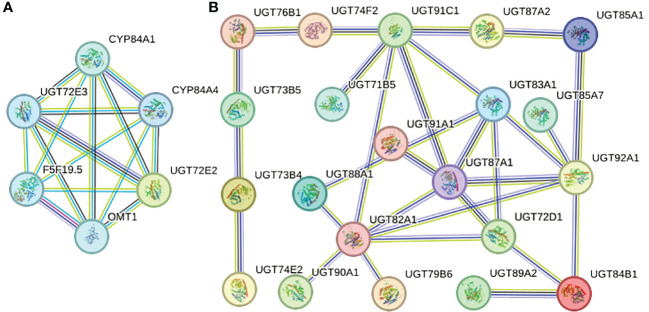
Protein-protein interaction network of specific PgUGT proteins. The color of the nodes represents different degrees of interaction, the thickness of each node line represents the strength of protein interaction.

Each AtUGT protein may have homology with multiple PgUGT proteins; therefore, we selected the PgUGT protein with the highest homology for analysis (PgUGT and AtUGT string proteins are listed in **Supplementary Table S3**). As shown in **Figure 6B**, 21 proteins had the characteristic of interaction and the same protein could interact with multiple proteins. For example, UGT87A1 (49.6% homology with PGrchr06G1265) could interact with UGT91A1 (50.0% homology with PGrchr07G0598), UGT91C1 (46.9% homology with PGrchr07G0646), UGT83A1 (42.0% homology with PGrchr02G2027), UGT92A1 (53.2% homology with PGrchr06G0150), UGT72D1 (48.5% homology with PGrchr01G1539), and UGT82A1 (51.9% homology with PGrchr05G0785). Therefore, we speculated that PGrchr06G1265 may interact with PGrchr07G0598, PGrchr07G0646, PGrchr02G2027, PGrchr06G0150, PGrchr01G1539, and PGrchr05G0785. Ten interaction proteins were co-expressed (Co-expression and combined scores are listed in **Supplementary Table S4**), among which the co-expression scores of UGT73B4 (41.9% homology with PGrchr03G1431) and UGT73B5 (46.7% homology with PGrchr03G1428) were highest, at 0.449, and the combined score was 0.423, indicating that these two proteins may have a strong interaction. Additionally, UGT72E2, UGT72E3 might interact with other proteins, like CYP84A1, CYP84A4, F5F19.5, and OMT1 (**Figure 6A**).

### Analysis of PgUGT expression patterns in the root, stem, and leaf

**Figure 7:**
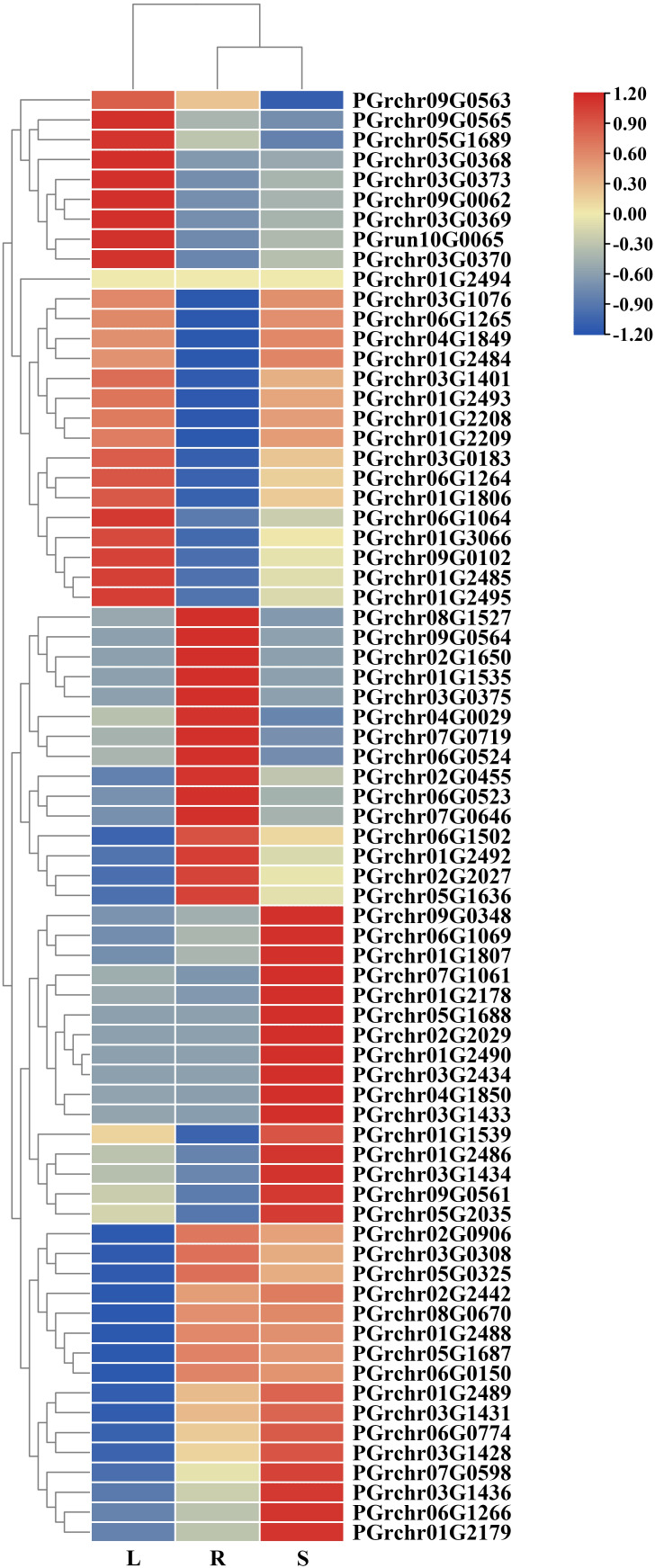
The expression of PgUGTs in the root, stem and leaf. The redder color represents the higher expression, and the bluer color represents the lower expression.

The expression of PgUGTs in the roots, stems, and leaves were analyzed using transcriptome-linked genome data (The fragments per kilobase of transcript per million mapped reads [FPKM] values are listed in **Supplementary Table S5**). Sixteen members showed high expression in the roots (FPKM >10), among which the FPKM value of PGrchr03G0308 was 248.72, showing the highest expression (FPKM>100). Meanwhile, 17 members were highly expressed in the leaf and 24 members were highly expressed in the stem, among which PGrchr03G0308 (FPKM = 149.27) and PGrchr09G0348 (FPKM = 120.51) showed the highest expression (**Figure 7**). In addition, seven members were highly expressed in the roots, stems, and leaves (PGrchr09G0348, PGrchr07G0719, PGrchr07G1061, PGrchr03G0308, PGrchr05G2035, PGrchr01G2492, and PGrchr01G3066), indicating that these seven members may play important roles in the development of the roots, stems, and leaves of *P. grandiflorus*. We chose 12 genes highly expressed in roots and verified the expression profiles of the 12 PgUGTs in the roots, stems, and leaves using qRT-PCR (Primer sequences are listed in **Supplementary Table S6**). The RNA-seq and qRT-PCR results revealed high consistency, indicating that the RNA-seq data were reliable and accurate (qRT-PCR results are presented in **Supplementary Figure S3**).

### Expression analysis of PgUGTs under drought stress

**Figure 8:**
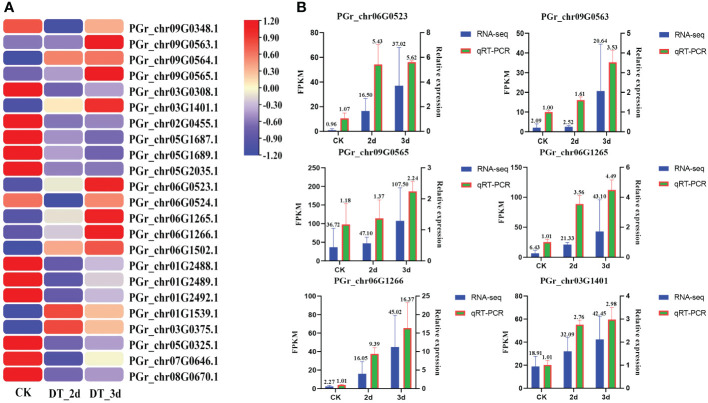
**(A)** The expression of PgUGTs under drought stress. The redder color represents the higher expression, and the bluer color represents the lower expression. Six genes were marked with star sharp. **(B)** qRT-PCR validation of six PgUGTs. The blue bars represent the FPKM values of genes from RNA-seq, and green bars represent the relative expression determined by qRT-PCR.

The expression patterns of PgUGTs under drought conditions were analyzed in this study. A total of 23 members showed significant changes in their expression levels (the FPKM values of 23 members are listed in **Supplementary Table S7**). Among these, six members (PGrchr09G0563, PGrchr09G0565, PGrchr03G1401, PGrchr06G0523, PGrchr06G1265, PGrchr06G1266) were obviously upregulated obviously after 3d of drought treatment (**Figure 8A**). Based on the above results, we speculated that the six PgUGTs whose expression levels were significantly upregulated under drought stress might play crucial roles in synthesizing platycodins. Therefore, we validated these six members using qRT-PCR (**Figure 8B**, Primer sequences are listed in **Supplementary Table S8**) and we selected three members (PGrchr09G0563, PGrchr06G0523, PGrchr06G1266) with great responses in the cloning and bioinformatics analyses. Among the three genes, PGrchr06G1266 expression was most substantially changed, with a 16.21-fold increase after 3d of drought treatment.

### Cloning and bioinformatics analysis of PGrchr06G0523, PGrchr06G1266, and PGrchr09G0563

**Figure 9:**
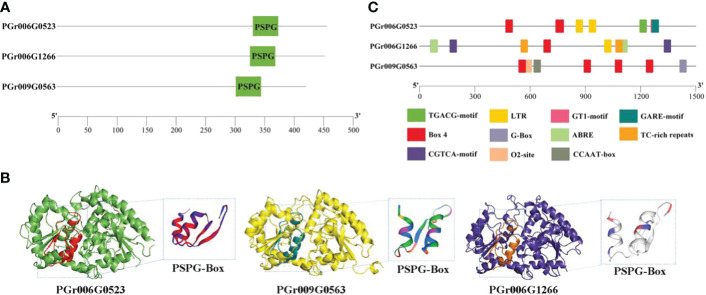
**(A)** Conserved motif PSPG-Box and its position of three genes. **(B)** 3D structure of three genes. **(C)** *Cis*-acting elements of three genes.

The candidate genes PGrchr06G0523 and PGrchr06G1266 were successfully cloned (Bacterial PCR results are presented in **Supplementary Figure S4**) and PGrchr09G0563 was performed to total gene synthesis. The three genes contained a highly conserved sequence (PSPG box) with 44 amino acids, which existed in the 300–400 aa range (**Figure 9A**). The three-dimensional (3D) structure of candidate UGTs and PSPG-box model is shown in **Figure 9B**. The protein model of PGrchr06G0523 was 5u6s.1 (George Thompson et al., 2017), sequence identity was 49.33% and the global model quality estimate (GMQE) was 0.78. The protein model of PGrchr09G0563 was A0A5B7BWI9, sequence identity was 74.94% and GMQE was 0.92. The protein model of PGrchr06G1266 was 6l8x.1.A (Li et al., 2020), sequence identity was 28.74% and GMQE was 0.68. Since the gene expression levels are regulated by promoters, the upstream 1500 bp promoter sequences of the three genes were examined, several *cis*-acting elements related to stress response exist in the promoter, including drought-inducibility element (MBS), low-temperature responsive element (LTR), MYB recognition site (MYB), light-response elements (I-box, GT1-motif, AT1-motif, TCT-motif, G-Box, Box 4), defense and stress responsiveness (TC-rich repeats), and other hormone-responsive elements (ABRE, TGACG-motif, GARE-motif) (**Supplementary Table S9**). The three genes both contained light-response elements, O2-site exited in PGr009G0563, MBS and LTR existed in PGr006G0523 and PGr006G1266, PGr006G1266 also contained TC-rich repeats, ABRE and TGACG-motif (**Figure 9C**), which means the PGr006G1266 gene played an important role in the response of *P. grandiflorus* to various stresses.

## Discussion

### Groups A and E of the PgUGT family make important contributions to the glycosylation of *P. grandiflorus* secondary metabolites

UGT families have been identified in several plant species. However, no further information has been reported about the UGT genes in *P. grandiflorus*. In this study, a total of 75 PgUGTs were identified and clustered into 14 groups, with Groups F and K not containing any members, and Groups P and O each having 5. Only one member was in group N, which was the same as that in dicotyledons, such as *A. thaliana* and peach. It provided additional evidence for the previous speculation that Group N was mainly amplified in monocotyledons (Li et al., 2014). Group F contained no PgUGTs. In contrast, dicotyledons, such as *A. thaliana* and *V. vinifera*, contained more members in Group F (Caputi et al., 2012). Group E contained nine UGT genes. Furthermore, the UGTs in Group E have been functionally identified in many plants (Song et al., 2016a, Song et al., 2016b; Dare et al., 2017), indicating that Group E members make crucial contributions to the glycosylation of *P. grandiflorus* secondary metabolites. Group A contained six UGT genes. PGr008G1527 and PGr003G2434 were clustered with PgGT1; this gene was confirmed to catalyze platycodin D to produce platycodin D3 and platycodin E (Tang et al., 2023). Hence, we infer that PGr008G1527 and PGr003G2434 may be able to catalyze the C-3 position of platycodin D in the same manner as PgGT1, and their enzymatic reactions may deserve further study.

### PgUGTs are expressed in a tissue-specific manner and are involved in the drought stress response

In plants, the expression levels of genes in various tissue locations reflects the strength of their roles in various tissues. For example, UGTs in cotton are highly expressed in the calli, seeds, fibers, and other developing tissues (Huang et al., 2015). In flax, UGT is highly expressed in the seeds and stems (Kraushofer and Sontag, 2002), causing seeds and stems to contain many secondary metabolites. Through tissue analysis of UGT expression patterns, we found the tissue-specific expression of PgUGT genes. Sixteen members were highly expressed in the root, while platycodin also mainly exists in the roots (Nyakudya et al., 2014), indicating that these genes might be related to platycodin synthesis. Seven members (PGrchr09G0348, PGrchr07G0719, PGrchr07G1061, PGrchr03G0308, PGrchr05G2035, PGrchr01G2492, and PGrchr01G3066) showed high expression in the roots, stems, and leaves, which means these genes may play significant roles in synthesizing secondary metabolites in these tissues. The myb-binding site (MBS) *cis*-element is reportedly important for drought stress responses (Xiong et al., 2022). In this study, the promoter regions of the 75 PgUGTs contained many response elements, including defense and stress responses (TC-rich repeats), low-temperature response (LTR), drought-inducibility (MBS) and other responses. Forty-five genes contained MBS, indicating their potential involvement in regulating *P. grandiflorus* response to drought stress. Additionally, expression patterns of the 75 PgUGTs were analyzed under drought conditions based on the RNA-seq results, and 23 differentially expressed PgUGTs were identified. According to the above results, many PgUGTs may be involved in the drought stress response.

### PGrchr06G1266 plays an important role in drought stress tolerance

UGTs play crucial roles in plant development processes, as well as abiotic stress responses (Ouyang et al., 2023). In our study, six UGT genes showed significant changes in response to drought stress. The developed real-time PCR assay showed three genes (PGrchr06G0523, PGrchr06G1266 and PGrchr09G0563) were highly responsive to drought stress, the PGrchr06G1266 gene expression was increased 16.21-fold after 3d of drought stress treatment. The *cis*-acting element in the promoter region induces gene expression, which is closely related to its biological function. Studies have shown that the presence of these *cis*-acting elements in UGTs may induce stress responses. AtUGT73B5 and AtUGT73B2 responded to oxidative stress (Mazel and Levine, 2002; Ahrazem et al., 2015), AtUGT74E2 responded to water stress (Tognetti et al., 2010), AtUGT84A1, AtUGT84A2, AtUGT84A3, and AtUGT84A4 responded to UV-B radiation stress (Schweiger et al., 2013). The three selected genes, PGrchr06G0523, PGrchr06G1266, and PGrchr09G0563, were cloned and their promoter sequences were characterized. Notably, the promoter sequence of three PgUGTs contain many action elements related to biological and abiotic stresses, such as light, temperature, drought, and hormone response elements. Among them, PGrchr06G1266 contains most response elements (21 cis-acting elements) including two TC-rich repeats (defense and stress responsiveness), two MYBs (MYB recognition site), one MBS (MYB binding site involved in drought-inducibility) and other response elements. The *cis*-acting elements and real-time PCR results indicated PGrchr06G1266 plays an important role in drought stress resistance.

## Conclusion

In the present study, 75 PgUGTs with a highly conserved sequence (PSPG box) were identified and divided into 14 groups. The promoter region contains response elements to light, hormones, drought stress, and other stimuli. Intraspecific collinearity analysis showed 22 pairs of tandemly duplicate genes, with 16, 17, and 24 genes being highly expressed in the roots, leaves, and stems, respectively. In addition, three genes were significantly expressed after 3d drought stress, especially PGr006G1266, which showed 16.21-fold greater expression after 3d of treatment. The three genes were cloned and promoter analysis was conducted; PGr006G1266 contained the most *cis*-elements to respond abiotic stress. This work may inspire further research on the molecular mechanisms of UGTs in synthesizing platycodins and controlling responses to drought stress.

## Data availability statement

The original contributions presented in the study are included in the article/supplementary material, further inquiries can be directed to the corresponding author/s. The UGT sequences of A. thaliana are available online (http://www.p450.kvl.dk/UGT.shtml).

## Author contributions

BC: Writing – original draft. XW: Writing – original draft. HY: Methodology, Writing – review & editing. ND: Methodology, Writing – review & editing. JiL: Methodology, Writing – review & editing. XWC: Data curation, Writing – review & editing. JW: Data curation, Writing – review & editing. CJ: Data curation, Writing – review & editing. JuL: Data curation, Writing – review & editing. XLC: Data curation, Writing – review & editing. LZ: Supervision, Writing – review & editing. SG: Funding acquisition, Supervision, Writing – review & editing.
