# Supplementary material for: Genome-wide analysis of UDP-glycosyltransferases family and identification of UGT genes involved in drought stress of Platycodon grandiflorus
Source: Front Plant Sci. 2024 Apr 29;15:1363251. doi: 10.3389/fpls.2024.1363251 (PMC11089202; doi:10.3389/fpls.2024.1363251)
Supplement: Supplementary file 1 [file DataSheet_1.docx]

Supplementary Materials

**Table.S1** Physical and chemical properties of PgUGTs*.*

| Gene id | Amino acids | Mw | pI | Subcellular localization |
| --- | --- | --- | --- | --- |
| PGr009G0062.1 | 484 | 53734.46 | 5.39 | Cell membrane |
| PGr009G0102.1 | 422 | 46641.40 | 4.93 | Chloroplast  Nucleus |
| PGr009G0348.1 | 682 | 75554.24 | 4.84 | Chloroplast |
| PGr009G0561.1 | 464 | 51912.61 | 5.33 | Cell membrane |
| PGr009G0563.1 | 424 | 47575.45 | 5.18 | Chloroplast |
| PGr009G0564.1 | 459 | 51741.23 | 5.42 | Cell membrane |
| PGr009G0565.1 | 462 | 52075.55 | 4.96 | Cell membrane |
| PGr007G0598.1 | 1000 | 112510.67 | 5.80 | Cell membrane |
| PGr007G0646.1 | 463 | 52019.54 | 5.03 | Cell membrane |
| PGr007G0719.1 | 483 | 54939.88 | 5.80 | Cell membrane  Chloroplast |
| PGr007G1061.1 | 462 | 52098.59 | 5.52 | Cell membrane |
| PGr003G0183.1 | 493 | 54347.26 | 6.09 | Cell membrane |
| PGr003G0308.1 | 486 | 54906.19 | 5.82 | Cell membrane  Chloroplast |
| PGr003G0368.1 | 524 | 58312.68 | 5.92 | Chloroplast |
| PGr003G0369.1 | 474 | 52990.67 | 6.06 | Chloroplast |
| PGr003G0370.1 | 477 | 53442.16 | 5.98 | Cell membrane  Chloroplast |
| PGr003G0373.1 | 477 | 53356.22 | 6.14 | Cell membrane  Chloroplast |
| PGr003G0375.1 | 463 | 52411.65 | 5.73 | Cell membrane  Chloroplast |
| PGr003G1076.1 | 408 | 45859.08 | 5.48 | Cell membrane  Chloroplast |
| PGr003G1401.1 | 471 | 53361.20 | 5.99 | Cell membrane  Chloroplast  Cytoplasmic  Nucleus |
| PGr003G1428.1 | 371 | 41466.79 | 5.10 | Cell membrane  Chloroplast |
| PGr003G1431.1 | 375 | 42169.50 | 5.66 | Cell membrane  Chloroplast |
| PGr003G1433.1 | 516 | 57358.62 | 5.61 | Cell membrane  Chloroplast |
| PGr003G1434.1 | 501 | 55779.97 | 5.68 | Cell membrane |
| PGr003G1436.1 | 222 | 24503.02 | 5.18 | Chloroplast |
| PGr003G2434.1 | 467 | 52632.45 | 5.20 | Cell membrane |
| PGr002G0455.1 | 427 | 48436.95 | 6.36 | Cell membrane |
| PGr002G0906.1 | 547 | 61113.37 | 6.40 | Chloroplast |
| PGr002G1650.1 | 499 | 56130.85 | 6.27 | Cell membrane  Chloroplast |
| PGr002G2027.1 | 456 | 50830.49 | 5.16 | Cell membrane |
| PGr002G2029.1 | 456 | 50694.49 | 5.57 | Cell membrane  Chloroplast |
| PGr002G2442.1 | 752 | 84002.01 | 6.50 | Cell membrane  Chloroplast  Cytoplasmic  Extracell  Mitochondrion  Nucleus  Vacuole |
| PGr005G0325.1 | 416 | 46777.29 | 5.12 | Vacuole |
| PGr005G0785.1 | 301 | 34078.10 | 7.57 | Chloroplast |
| PGr005G1636.1 | 270 | 30871.32 | 5.05 | Chloroplast |
| PGr005G1687.1 | 487 | 54789.84 | 5.74 | Cell membrane  Chloroplast |
| PGr005G1688.1 | 487 | 54947.09 | 5.70 | Chloroplast |
| PGr005G1689.1 | 604 | 67566.95 | 4.79 | Chloroplast |
| PGr005G2035.1 | 487 | 54700.56 | 5.76 | Cell membrane  Chloroplast |
| PGr008G0670.1 | 459 | 51278.06 | 6.11 | Cell membrane |
| PGr008G1527.1 | 463 | 52337.52 | 5.70 | Cell membrane |
| PGr006G0150.1 | 436 | 49443.40 | 5.24 | Cell membrane  Chloroplast  Cytoplasmic  Nucleus  Peroxisome |
| PGr006G0523.1 | 462 | 51288.07 | 4.85 | Cell membrane |
| PGr006G0524.1 | 455 | 50628.93 | 5.30 | Cell membrane |
| PGr006G0774.1 | 431 | 48464.84 | 5.73 | Cell membrane  Chloroplast |
| PGr006G1064.1 | 475 | 53414.16 | 6.29 | Cell membrane |
| PGr006G1069.1 | 454 | 51022.59 | 6.11 | Cell membrane |
| PGr006G1264.1 | 462 | 51479.31 | 5.89 | Chloroplast |
| PGr006G1265.1 | 456 | 50961.52 | 5.71 | Cell membrane  Chloroplast |
| PGr006G1266.1 | 456 | 51102.56 | 5.57 | Cell membrane  Chloroplast |
| PGr006G1502.1 | 449 | 50653.76 | 5.98 | Cell membrane  Chloroplast |
| PGr004G0029.1 | 458 | 50677.87 | 5.58 | Cell membrane  Chloroplast |
| PGr004G0030.1 | 492 | 54233.21 | 6.51 | Cell membrane  Chloroplast |
| PGr004G1849.1 | 483 | 54066.20 | 5.61 | Cell membrane |
| PGr004G1850.1 | 375 | 41975.31 | 6.22 | Chloroplast |
| PGr001G1535.1 | 275 | 31107.13 | 7.69 | Chloroplast |
| PGr001G1539.1 | 553 | 61691.83 | 6.07 | Chloroplast |
| PGr001G1806.1 | 477 | 52931.63 | 5.25 | Cell membrane  Chloroplast |
| PGr001G1807.1 | 487 | 54425.55 | 5.84 | Cell membrane  Cytoplasmic |
| PGr001G2178.1 | 462 | 52316.29 | 5.93 | Cell membrane |
| PGr001G2179.1 | 456 | 50729.64 | 5.46 | Cell membrane |
| PGr001G2208.1 | 453 | 49485.83 | 6.13 | Cell membrane  Chloroplast |
| PGr001G2209.1 | 496 | 55545.92 | 6.23 | Chloroplast |
| PGr001G2484.1 | 466 | 52225.12 | 5.77 | Cell membrane |
| PGr001G2485.1 | 464 | 52404.28 | 5.52 | Cell membrane  Chloroplast |
| PGr001G2486.1 | 464 | 52262.86 | 5.77 | Cell membrane  Chloroplast |
| PGr001G2488.1 | 475 | 52623.72 | 5.68 | Chloroplast |
| PGr001G2489.1 | 470 | 52574.85 | 5.23 | Cell membrane  Chloroplast |
| PGr001G2490.1 | 260 | 28737.01 | 5.10 | Cell membrane  Chloroplast  Peroxisome |
| PGr001G2492.1 | 464 | 51887.55 | 5.81 | Cell membrane  Chloroplast |
| PGr001G2493.1 | 545 | 61006.43 | 5.85 | Cell membrane  Chloroplast |
| PGr001G2494.1 | 330 | 37008.56 | 5.92 | Chloroplast |
| PGr001G2495.1 | 508 | 56839.59 | 5.52 | Cell membrane  Chloroplast |
| PGr001G3066.1 | 986 | 108458.17 | 5.40 | Cell membrane  Chloroplast  Nucleus |
| PGrun10G0065.1 | 484 | 53838.63 | 5.39 | Cell membrane  Chloroplast |

**Table.S2** The homology of 22 pairs of tandemly duplicated genes.

|  | Chromosome | ID | | Homology(%) |
| --- | --- | --- | --- | --- |
| Pair_1 | Chr01 | PGr001G1806 | PGr001G1807 | 63.64 |
| Pair_2 | Chr01 | PGr001G2178 | PGr001G2179 | 66.02 |
| Pair_3 | Chr01 | PGr001G2208 | PGr001G2209 | 51.99 |
| Pair_4 | Chr01 | PGr001G2484 | PGr001G2485 | 69.96 |
| Pair_5 | Chr01 | PGr001G2485 | PGr001G2486 | 79.31 |
| Pair_6 | Chr01 | PGr001G2488 | PGr001G2489 | 80.75 |
| Pair_7 | Chr01 | PGr001G2489 | PGr001G2490 | 41.28 |
| Pair_8 | Chr01 | PGr001G2492 | PGr001G2493 | 27.61 |
| Pair_9 | Chr01 | PGr001G2493 | PGr001G2494 | 33.51 |
| Pair_10 | Chr01 | PGr001G2494 | PGr001G2495 | 30.00 |
| Pair_11 | Chr03 | PGr003G0368 | PGr003G0369 | 88.57 |
| Pair_12 | Chr03 | PGr003G0369 | PGr003G0370 | 97.91 |
| Pair_13 | Chr03 | PGr003G1433 | PGr003G1434 | 69.84 |
| Pair_14 | Chr04 | PGr004G0029 | PGr004G0030 | 57.63 |
| Pair_15 | Chr04 | PGr004G1849 | PGr004G1850 | 71.22 |
| Pair_16 | Chr05 | PGr005G1687 | PGr005G1688 | 52.52 |
| Pair_17 | Chr05 | PGr005G1688 | PGr005G1689 | 46.48 |
| Pair_18 | Chr06 | PGr006G0523 | PGr006G0524 | 87.88 |
| Pair_19 | Chr06 | PGr006G1264 | PGr006G1265 | 65.02 |
| Pair_20 | Chr06 | PGr006G1265 | PGr006G1266 | 97.15 |
| Pair_21 | Chr09 | PGr009G0563 | PGr009G0564 | 86.12 |
| Pair_22 | Chr09 | PGr009G0564 | PGr009G0565 | 94.82 |

**Table.S3** PgUGT and AtUGT string proteins.

| Gene ID (PgUGTs) | String protein (AtUGTs) | Identity(%) |
| --- | --- | --- |
| PGrchr01G2209 | UGT71B5 | 30.0 |
| PGrchr01G1539 | UGT72D1 | 48.5 |
| PGrchr03G1431 | UGT73B4 | 41.9 |
| PGrchr03G1428 | UGT73B5 | 46.7 |
| PGrchr09G0561 | UGT74E2 | 48.9 |
| PGrchr09G0564 | UGT74F2 | 56.1 |
| PGrchr01G2484 | UGT76B1 | 52.6 |
| PGrchr02G2442 | UGT79B6 | 51.4 |
| PGrchr05G0785 | UGT82A1 | 51.9 |
| PGrchr02G2027 | UGT83A1 | 42.0 |
| PGrchr03G1401 | UGT84B1 | 46.2 |
| PGrchr09G0062 | UGT85A1 | 59.3 |
| PGrchr05G1636 | UGT85A7 | 43.4 |
| PGrchr06G1265 | UGT87A1 | 49.6 |
| PGrchr06G1264 | UGT87A2 | 53.0 |
| PGrchr01G2208 | UGT88A1 | 28.5 |
| PGrchr02G0906 | UGT89A2 | 48.8 |
| PGrchr05G0325 | UGT90A1 | 53.8 |
| PGrchr07G0598 | UGT91A1 | 50.0 |
| PGrchr07G0646 | UGT91C1 | 46.9 |
| PGrchr06G0150 | UGT92A1 | 53.2 |

**Table.S4** Cooccurrence across genomes scores, co-expression sores and combined scores of interaction proteins.

| Interaction proteins | Cooccurrence Across Genomes | Co-Expression | Combined Score |
| --- | --- | --- | --- |
| UGT83A1 and UGT88A1 | 0.201 | 0.449 | 0.419 |
| UGT83A1 and UGT87A1 | 0.175 | 0.206 | 0.401 |
| UGT83A1 and UGT72D1 | 0.211 | 0.199 | 0.449 |
| UGT87A1 and UGT91A1 | 0.176 | 0.138 | 0.404 |
| UGT87A1 and UGT82A1 | 0.186 | 0.103 | 0.444 |
| UGT87A1 and UGT72D1 | 0.199 | 0.099 | 0.43 |
| UGT88A1 and UGT82A1 | 0.155 | 0.061 | 0.402 |
| UGT82A1 and UGT72D1 | 0.141 | 0.058 | 0.402 |
| UGT82A1 and UGT90A1 | 0.153 | 0.056 | 0.454 |
| UGT82A1 and UGT79B6 | 0.14 | 0.042 | 0.412 |
| UGT74E2 and UGT73B4 | 0.154 | - | 0.423 |
| UGT73B4 and UGT73B5 | 0.048 | - | 0.461 |
| UGT73B5 and UGT76B1 | 0.173 | - | 0.407 |
| UGT76B1 and UGT74F2 | 0.167 | - | 0.426 |
| UGT74F2 and UGT91C1 | 0.204 | - | 0.404 |
| UGT91C1 and UGT71B5 | 0.188 | - | 0.42 |
| UGT91C1 and UGT82A1 | 0.191 | - | 0.433 |
| UGT91C1 and UGT87A1 | 0.207 | - | 0.452 |
| UGT91C1 and UGT83A1 | 0.187 | - | 0.401 |
| UGT91C1 and UGT87A2 | 0.226 | - | 0.417 |
| UGT87A2 and UGT85A1 | 0.142 | - | 0.415 |
| UGT85A1 and UGT92A1 | 0.205 | - | 0.423 |
| UGT92A1 and UGT85A7 | 0.202 | - | 0.405 |
| UGT92A1 and UGT83A1 | 0.209 | - | 0.464 |
| UGT92A1 and UGT87A1 | 0.187 | - | 0.414 |
| UGT92A1 and UGT82A1 | 0.136 | - | 0.437 |
| UGT92A1 and UGT84B1 | 0.211 | - | 0.415 |
| UGT84B1 and UGT72D1 | 0.200 | - | 0.406 |
| UGT84B1 and UGT89A2 | 0.177 | - | 0.432 |

**Table.S5** The expression of PgUGTs in the roots, stems, and leaves.

| Gene ID | R | S | L |
| --- | --- | --- | --- |
| PGrchr09G0062 | 0.00 | 0.27 | 3.77 |
| PGrchr09G0102 | 0.23 | 4.65 | 33.49 |
| PGrchr09G0348 | 44.31 | 120.51 | 38.56 |
| PGrchr09G0561 | 0.00 | 0.16 | 0.05 |
| PGrchr09G0563 | 1.17 | 0.22 | 1.83 |
| PGrchr09G0564 | 7.50 | 0.29 | 0.28 |
| PGrchr09G0565 | 3.30 | 2.15 | 19.98 |
| PGrchr07G0719 | 52.60 | 10.85 | 13.71 |
| PGrchr07G1061 | 11.39 | 20.71 | 12.12 |
| PGrchr03G0183 | 1.66 | 16.18 | 41.65 |
| PGrchr03G0308 | 248.72 | 149.27 | 17.13 |
| PGrchr03G1076 | 0.00 | 1.47 | 1.53 |
| PGrchr03G1401 | 0.45 | 22.60 | 51.04 |
| PGrchr02G0455 | 95.65 | 6.57 | 1.82 |
| PGrchr02G2027 | 26.67 | 13.86 | 7.63 |
| PGrchr02G2029 | 0.00 | 0.18 | 0 |
| PGrchr02G2442 | 7.00 | 8.28 | 1.41 |
| PGrchr05G1636 | 20.91 | 10.34 | 5.7 |
| PGrchr05G1687 | 43.16 | 33.41 | 0.14 |
| PGrchr05G1688 | 0.23 | 1.05 | 0.23 |
| PGrchr05G1689 | 0.71 | 0.40 | 1.89 |
| PGrchr05G2035 | 11.97 | 59.06 | 21.95 |
| PGrchr06G0523 | 0.96 | 0.24 | 0.15 |
| PGrchr06G0524 | 1.43 | 0.04 | 0.2 |
| PGrchr06G0774 | 5.11 | 9.73 | 1.17 |
| PGrchr06G1064 | 0.03 | 0.16 | 0.47 |
| PGrchr06G1069 | 1.17 | 3.63 | 0.85 |
| PGrchr06G1264 | 1.66 | 16.71 | 54.4 |
| PGrchr06G1265 | 2.72 | 18.65 | 19.38 |
| PGrchr06G1266 | 0.28 | 0.93 | 0.11 |
| PGrchr06G1502 | 9.38 | 4.06 | 0.82 |
| PGrchr04G0029 | 5.62 | 0.31 | 0.95 |
| PGrchr04G1849 | 0.00 | 0.29 | 0.28 |
| PGrchr04G1850 | 4.51 | 72.58 | 4.89 |
| PGrchr01G1535 | 0.03 | 0.00 | 0 |
| PGrchr01G1806 | 0.14 | 2.64 | 5.87 |
| PGrchr01G1807 | 4.47 | 15.04 | 3.41 |
| PGrchr01G2178 | 0.34 | 2.64 | 0.45 |
| PGrchr01G2179 | 0.05 | 0.20 | 0 |
| PGrchr01G2208 | 0.60 | 23.11 | 33.98 |
| PGrchr01G2209 | 0.90 | 14.57 | 18.82 |
| PGrchr01G2484 | 0.47 | 3.28 | 3.09 |
| PGrchr01G2485 | 0.02 | 0.28 | 0.74 |
| PGrchr01G2486 | 0.04 | 1.75 | 0.34 |
| PGrchr01G2488 | 2.30 | 2.23 | 0.62 |
| PGrchr01G2489 | 3.89 | 6.94 | 0.39 |
| PGrchr01G2490 | 0.00 | 0.92 | 0 |
| PGrchr01G2492 | 90.53 | 25.70 | 10.73 |
| PGrchr01G2493 | 2.40 | 6.28 | 7.42 |
| PGrchr01G2494 | 0.00 | 0.00 | 0 |
| PGrchr01G2495 | 2.25 | 6.26 | 22.83 |
| PGrchr01G3066 | 10.03 | 29.78 | 79.07 |
| PGrun10G0065 | 0.00 | 0.28 | 2.53 |
| PGrchr01G1539 | 1.74 | 63.53 | 17.89 |
| PGrchr02G0906 | 17.59 | 15.85 | 8.07 |
| PGrchr02G1650 | 0.07 | 0.00 | 0 |
| PGrchr03G0368 | 0.00 | 0.03 | 0.54 |
| PGrchr03G0369 | 0.05 | 0.18 | 1.21 |
| PGrchr03G0370 | 0.05 | 0.47 | 3.46 |
| PGrchr03G0373 | 0.00 | 0.20 | 2.05 |
| PGrchr03G0375 | 4.31 | 0.00 | 0 |
| PGrchr03G1428 | 0.09 | 0.11 | 0.06 |
| PGrchr03G1431 | 1.20 | 1.73 | 0.22 |
| PGrchr03G1433 | 0.27 | 1.46 | 0.3 |
| PGrchr03G1434 | 0.12 | 2.38 | 0.45 |
| PGrchr03G1436 | 1.42 | 12.38 | 0 |
| PGrchr03G2434 | 0.00 | 0.02 | 0 |
| PGrchr05G0325 | 15.48 | 13.18 | 6.83 |
| PGrchr06G0150 | 18.75 | 16.45 | 1 |
| PGrchr07G0598 | 0.19 | 0.43 | 0.02 |
| PGrchr07G0646 | 4.34 | 0.28 | 0 |
| PGrchr08G0670 | 26.72 | 27.89 | 2.87 |
| PGrchr08G1527 | 29.11 | 0.33 | 0.64 |

**Table.S6** Primer used for quantification of transcripts by means of real time quantitative PCR

| Gene id | Forward primer (5’ to 3’) | Reward primer(5’ to 3’) |
| --- | --- | --- |
| PGrchr01G2492 | CCTCCAAGCACCCAACTCA | CAGGTCGAACCACCCACAA |
| PGrchr01G3066 | CGGGTTTGCCGCCTATCTA | TGCTGATGAAGTTTGCGTAAGT |
| PGrchr02G0455 | TGGACGAATCCGAGTTTGTG | CCACCGCGTCTAAGAAACCA |
| PGrchr02G2027 | TTCCCAGAAGGATTTACCGTG | CCTGCAATGGAAGGGTGAG |
| PGrchr03G0308 | AATGGCAAGCGAGAAGCG | GCGCCCACCTTACGATAAA |
| PGrchr05G0325 | GAGGATGGTGGTGGAGGAGATT | TTCTCCAACCCTTGCCATTTC |
| PGrchr05G1636 | CTGGTGCCCTCAACAACAAG | TCTCTTCCTCCATCAACTCCC |
| PGrchr05G1687 | TTCATCGGTTGGAGGGTTCT | GCACAGGCATATCGGCAATT |
| PGrchr05G2035 | TCCAAAATGAGTCGAGCGTC | GGACCAAGGAGAATCAGCACG |
| PGrchr07G0719 | CGAATAGCAAGCACCCGTTT | TCCCAGAAACCTTCGAGCAA |
| PGrchr07G1061 | CATCGGGCAGTTGGTTGTTT | TGATCGGCCCACTGAGGTA |
| PGrchr08G1527 | TGAGTCATTGTGGGTGGAGTTC | CTTGCATTGAATGGCTGGTC |

**Table.S7** The FPKM value of 23 PgUGTs under drought stress

| Gene id | CK | DT-2d | DT-3d |
| --- | --- | --- | --- |
| PGrchr09G0348.1 | 246.94 | 53.98 | 204.80 |
| PGrchr09G0563.1 | 2.09 | 2.52 | 20.64 |
| PGrchr09G0564.1 | 12.72 | 37.18 | 38.06 |
| PGrchr09G0565.1 | 36.72 | 47.10 | 107.50 |
| PGrchr03G0308.1 | 92.66 | 12.42 | 25.18 |
| PGrchr03G1401.1 | 18.91 | 32.09 | 42.45 |
| PGrchr02G0455.1 | 24.43 | 0.91 | 1.95 |
| PGrchr05G1687.1 | 44.33 | 18.70 | 15.81 |
| PGrchr05G1689.1 | 79.47 | 44.47 | 37.67 |
| PGrchr05G2035.1 | 56.98 | 21.40 | 20.48 |
| PGrchr06G0523.1 | 0.96 | 16.50 | 37.02 |
| PGrchr06G0524.1 | 23.55 | 3.85 | 22.44 |
| PGrchr06G1265.1 | 6.43 | 21.33 | 43.10 |
| PGrchr06G1266.1 | 2.27 | 16.05 | 45.02 |
| PGrchr06G1502.1 | 33.94 | 105.19 | 123.44 |
| PGrchr01G2488.1 | 17.64 | 2.22 | 6.38 |
| PGrchr01G2489.1 | 65.98 | 5.61 | 26.27 |
| PGrchr01G2492.1 | 70.31 | 6.25 | 23.01 |
| PGrchr01G1539.1 | 19.08 | 45.67 | 38.07 |
| PGrchr03G0375.1 | 25.52 | 59.59 | 49.93 |
| PGrchr05G0325.1 | 49.69 | 5.12 | 12.07 |
| PGrchr07G0646.1 | 38.99 | 11.62 | 24.54 |
| PGrchr08G0670.1 | 30.01 | 0.23 | 3.99 |

**Table.S8** Primer sequences of six genes.

| Gene id | Forward primer (5’ to 3’) | Reward primer(5’ to 3’) |
| --- | --- | --- |
| PGrchr09G0563 | GACCAACAATCCCATCTTTCTACTT | ATTACGGAACCAGTCGGCTTAG |
| PGrchr09G0565 | GGACCAACAATCCCATCTTTCTAC | ATTTGCCATACTCCCGAATGATAC |
| PGrchr03G1401 | ATTAGGCCCGTTGGTCCATTA | GAGGAAGGCTGCTGTTTGTTTA |
| PGrchr06G0523 | GGACCAACAGTTCCCTCATTCTAC | GTACTTGACATATGCACCGAGATTC |
| PGrchr06G1265 | TCCATCACTATCATCTGCTTGTTCA | ATGTAGTCCACCAGCTCTTCTCCTT |
| PGrchr06G1266 | TCTATGGCATTGGTCAAGAGGTT | AATGGAGTAGACGGGGATTTGA |

**Table.S9** *Cis*-elements in the promoter region (~1.5 kb) of PGr006G0523, PGr006G1266 and PGr009G0563.

| GENE ID | Site Name | Sequence | Position | Strand | Function |
| --- | --- | --- | --- | --- | --- |
| PGr006G0523 | TGACG-motif | CGTCA | -1214 | - | MeJA-responsiveness |
| PGr006G0523 | MYB | CAACTG | -1429 | - | MYB recognition site |
| PGr006G0523 | MYB | CAACAG | -433 | + | MYB recognition site |
| PGr006G0523 | MYB | TAACCA | -1276 | + | MYB recognition site |
| PGr006G0523 | MYB | CAACAG | -1280 | + | MYB recognition site |
| PGr006G0523 | MYB | CAACCA | -1341 | + | MYB recognition site |
| PGr006G0523 | MYB | CAACCA | -1481 | + | MYB recognition site |
| PGr006G0523 | MBS | CAACTG | -1429 | - | MYB binding site involved in drought-inducibility |
| PGr006G0523 | I-Box | TGATAATGT | -572 | + | part of a light responsive element |
| PGr006G0523 | LTR | CCGAAA | -868 | + | low-temperature responsiveness |
| PGr006G0523 | LTR | CCGAAA | -939 | + | low-temperature responsiveness |
| PGr006G0523 | GT1-motif | GGTTAAT | -1274 | - | light responsive element |
| PGr006G0523 | GT1-motif | GGTTAA | -1275 | - | light responsive element |
| PGr006G0523 | TCT-motif | TCTTAC | -153 | - | part of a light responsive element |
| PGr006G0523 | TCT-motif | TCTTAC | -546 | + | part of a light responsive element |
| PGr006G0523 | W Box | TTGACC | -806 | - |  |
| PGr006G0523 | GARE-motif | TCTGTTG | -1280 | - | gibberellin-responsive element |
| PGr006G0523 | AT1-motif | AATTATTTTTTATT | -779 | + | part of a light responsive module |
| PGr006G0523 | TGACG-motif | TGACG | -1214 | + | MeJA-responsiveness |
| PGr006G0523 | Box 4 | ATTAAT | -486 | + | part of light responsiveness |
| PGr006G0523 | Box 4 | ATTAAT | -758 | - | part of light responsiveness |
| PGr006G0523 | Box 4 | ATTAAT | -764 | - | part of light responsiveness |
| PGr006G0523 | GA-motif | ATAGATAA | -977 | + | part of a light responsive element |
| PGr006G0523 | DRE core | GCCGAC | -947 | - | Dehydration-responsive element |
| PGr006G1266 | Gap-Box | CAAATGAA(A/G)A | -1218 | - | part of a light responsive element |
| PGr006G1266 | AT1-motif | AATTATTTTTTATT | -708 | + | part of a light responsive module |
| PGr006G1266 | W Box | TTGACC | -734 | - |  |
| PGr006G1266 | G-Box | GCCACGTGGA | -77 | - | light responsiveness |
| PGr006G1266 | G-Box | CACGTG | -79 | + | light responsiveness |
| PGr006G1266 | G-Box | CACGTG | -79 | + | light responsiveness |
| PGr006G1266 | G-Box | CACGTT | -1110 | + | light responsiveness |
| PGr006G1266 | ABRE | CACGTG | -79 | + | abscisic acid responsiveness |
| PGr006G1266 | ABRE | ACGTG | -80 | + | abscisic acid responsiveness |
| PGr006G1266 | ABRE | ACGTG | -1110 | - | abscisic acid responsiveness |
| PGr006G1266 | Box 4 | ATTAAT | -693 | + | part of light responsiveness |
| PGr006G1266 | TGACG-motif | TGACG | -183 | - | MeJA-responsiveness |
| PGr006G1266 | TGACG-motif | TGACG | -1346 | - | MeJA-responsiveness |
| PGr006G1266 | TC-rich repeats | ATTCTCTAAC | -568 | - | defense and stress responsiveness |
| PGr006G1266 | TC-rich repeats | GTTTTCTTAC | -1083 | - | defense and stress responsiveness |
| PGr006G1266 | LTR | CCGAAA | -1022 | + | low-temperature responsiveness |
| PGr006G1266 | MBS | CAACTG | -1481 | + | MYB binding site involved in drought-inducibility |
| PGr006G1266 | CGTCA-motif | CGTCA | -183 | + | MeJA-responsiveness |
| PGr006G1266 | CGTCA-motif | CGTCA | -1346 | + | MeJA-responsiveness |
| PGr006G1266 | MYB | TAACTG | -446 | - | MYB recognition site |
| PGr006G1266 | MYB | CAACTG | -1481 | + | MYB recognition site |
| PGr009G0563 | O2-site | GATGACATGG | -590 | - | zein metabolism regulation |
| PGr009G0563 | MYB | CAACCA | -578 | - | MYB recognition site |
| PGr009G0563 | MYB | CAACCA | -1056 | - | MYB recognition site |
| PGr009G0563 | CCAAT-Box | CAACGG | -639 | - | MYBHv1 binding site |
| PGr009G0563 | Box 4 | ATTAAT | -557 | + | part of light responsiveness |
| PGr009G0563 | Box 4 | ATTAAT | -911 | - | part of light responsiveness |
| PGr009G0563 | Box 4 | ATTAAT | -1080 | - | part of light responsiveness |
| PGr009G0563 | Box 4 | ATTAAT | -1249 | - | part of light responsiveness |
| PGr009G0563 | W Box | TTGACC | -48 | + |  |
| PGr009G0563 | G-Box | CACGAC | -1431 | - | light responsiveness |

**Figure.S1** Comparison results of PgGT1 and PGr008G1527 amino acids.

**

**

**Figure.S2** Conserved motif PSPG-Box of PgUGTs.


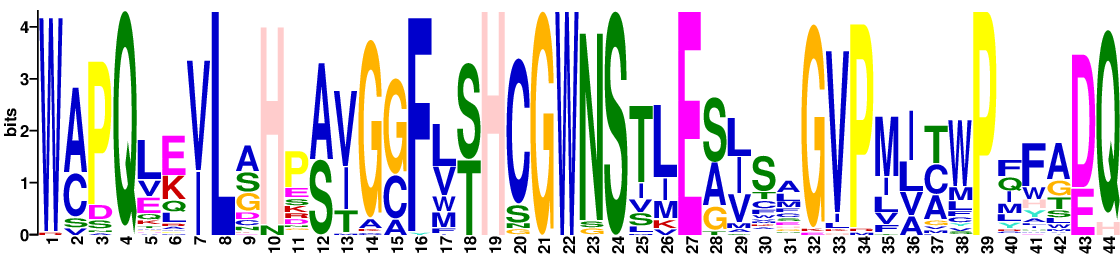


**Figure.S3** qRT-PCR validation of 12 PgUGTs. The blue lines represent the FPKM values of genes from RNA-seq, and green bars represent the relative expression determined by qRT-PCR.

**Figure.S4** Bacterial PCR results of PGrchr06G0523 (1389bp) and PGrchr06G1266 (1371bp).


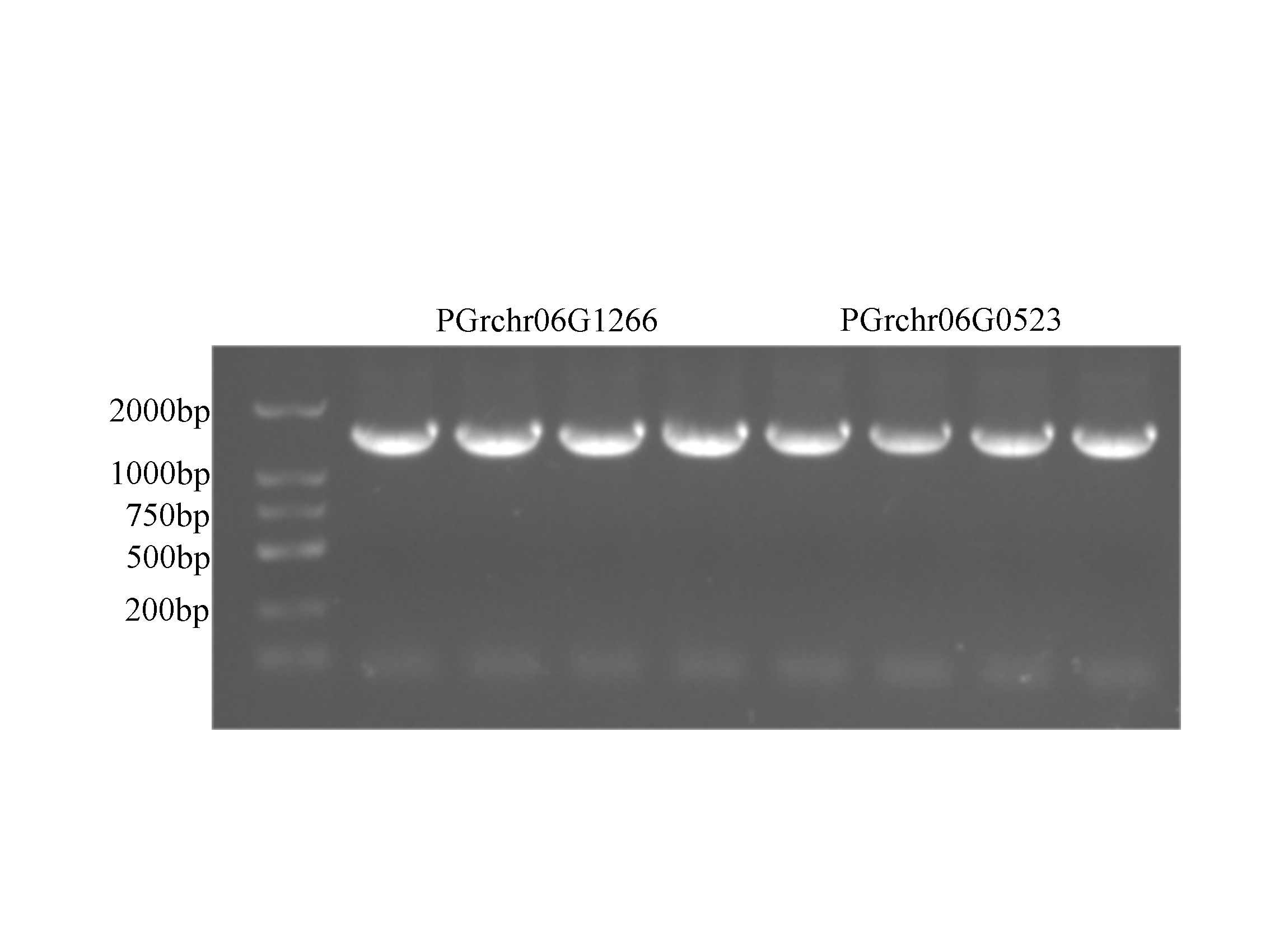


There are a total of 8 bands in the Figure.S4. The four on the left side represent 1266 bacterial liquid PCR bands, the right side represent 0523 bacterial liquid PCR bands.
